# The Treatment of Whooping Cough

**Published:** 1891-05

**Authors:** 


### THE TREATMENT OF WHOOPING COUGH.

The following treatment is used very largely by certain of the leading specialists in diseases of children in Paris, in cases of whooping-cough. It is divided into three periods. The patient should remain in one room or in bed, and the physician employs belladonna and small doses of opium with aconite, as in the following prescription:

| | |
|---|---|
| Tincture of aconite<br>Tincture of belladona<br>Camphorated tincture of opium | of each 1 drachm. |

Two to five drops once or twice a day, according to the age of the child, is the proper dose. If there is no febrile movement the amount of the aconite can be much decreased, and if constipation is present the opium should not be used. In the second period, or when vomiting comes on, ipecac may be given in small amounts to allay gastric irritation, and in the third period, when convalescence is established, cod liver oil, tonics, and Fowler's solution will be found of service.—*Med. News.*

PASTILS FOR FETID BREATH.—

℞ Ground roast coffee, 75 grammes.
Pulverized charcoal 25 grammes.
Pulverized boracic acid, 25 grammes.
Saccharine, 0 gram., 65 centigr.
Tincture of vanella, q. s.
Gum mucilage, q. s.
Make into pastils of 0 gram. 70 centigr. each.

